# The Number of Roots and Canals in the Maxillary Second Premolars in a Group of Jordanian Population

**DOI:** 10.1155/2014/797692

**Published:** 2014-11-03

**Authors:** Muna M. F. Al-Ghananeem, Khattar Haddadin, Abeer Salem Al-Khreisat, Moeen Al-Weshah, Nidal Al-Habahbeh

**Affiliations:** ^1^Conservative Dentistry Department, Royal Medical Services Hospitals, P.O. Box 536, Amman 11953, Jordan; ^2^Endodontics Department, Royal Medical Services Hospitals, P.O. Box 536, Amman 11953, Jordan

## Abstract

*Objectives*. The aim of this study was to investigate the number of roots and root canals in the maxillary second premolar in a group of Jordanian population. *Materials and Methods*. A total of 217 patients, 100 female (46%) and 117 male (54%), received root canal treatment of maxillary second premolar from January 2012 to January 2014. The mean age of the patients was 32.7, ranging from 18 to 60 years. The teeth included in the study were examined clinically and radiographically for the number of roots and root canals using magnifying loupes. *Results*. Out of the total of 217 maxillary second premolars, 120 teeth had one root (55.3%), 96 teeth had two roots (44.2%), and one tooth had three roots (0.46%). Regarding root canal configuration, 30 teeth (13.8%) had one canal, 54 teeth (24.9%) had two canals shared in one apical foramen, 132 teeth (60.8%) had two canals with two separate apical foramina, and one tooth (0.46%) had three canals with separate apical foramina. *Conclusion*. The incidence of two canals (either with shared or separate apical foramina) is very high in the maxillary second premolars in Jordanian population; therefore inspection should be done for the presence of second canal whenever endodontic treatment is planned for this tooth.

## 1. Introduction

A thorough knowledge of the anatomy of the tooth and morphology of the root canal is essential for the success of root canal treatment [1]. The ultimate goal of root canal treatment is a thorough cleaning and shaping of all pulp spaces and the complete obturation of these spaces with an inert filling material [1, 2]. The inadequate knowledge can lead to inadequate biomechanical instrumentation of root canal system, and this will cause failure of endodontic treatment [3].

It is well known that tooth anatomy varies according to racial origin [2–4]. Therefore it is very important to be familiar with variations in tooth anatomy and characteristic features in various racial groups since such knowledge can aid in location and negotiation of canals, as well as their subsequent management [5].

Review of the literature showed a high variability in the root canal morphology of the maxillary second premolar [6–9]. The aim of this study was to investigate clinically and radiographically the number of roots and root canals in the maxillary second premolar in a group of Jordanian population in routine endodontic practice using magnifying loupes.

## 2. Materials and Methods

Two hundred seventeen patients were referred to the Conservative Clinic at King Hussein Medical Centre for endodontic treatment of maxillary second premolar from January 2012 till January 2014. Preoperative radiographs were taken for evaluation (root morphology, number of canals, and periapical status). The teeth that were included in the study were those teeth that required nonsurgical endodontic treatment. The included teeth were free of root resorption, having no calcifications or open apices. No retreatment cases were included in the study.

Two radiographs in two planes were taken during routine endodontic treatment for these teeth (parallel and cone shift technique).

The endodontic procedures undertaken were as follows: local anesthesia (Ubistesin Forte/3M ESPE, Seefeld, Germany) was administered. Under rubber dam isolation an oval access cavity was opened between the cusp tips, being wider bucco-palatally, with sterile high and low speed burs with water coolant. After the contents of the pulp chamber were removed, a sharp endodontic explorer was used to explore the developmental grooves carefully to locate the orifices of the canals. Copious amount of 2.5% sodium hypochlorite irrigation was used. Pulp tissue was extirpated using barbed broaches (Nerve Broaches/Alfred Becht-GmbH, Germany) or H-Files (Mani Inc., Japan) and the canals were flared with gates glidden drills of numbers 2, 3, and 4 (Mani Inc., Japan). Two periapical radiographs in two angles were taken (parallel and cone shift technique) for evaluation of the number of roots and root canals as well as for confirmation of the working length after inserting size of 15, 20, or 25 K files (Mani Inc., Japan) in the canals. Examination of the floor of the pulp chamber to locate canals orifices is done using 3.5 high resolution magnification loupes (Keeler Inc., UK). The teeth included were both clinically and radiographically examined by two specialists with more than 10 years of experience in endodontics. The number of roots and root canals in the maxillary second premolars was recorded.

## 3. Results

A total of 217 patients 100 female (46%) and 117 male (54%) received root canal treatment of maxillary second premolar. The mean age of the patients was 32.7, ranging from 18 to 60 years.

Out of the total of 217 maxillary second premolars 120 teeth had one root (55.3%), 96 teeth had two roots (44.2%), and one tooth had three roots (0.46%).

**Figure 1:**
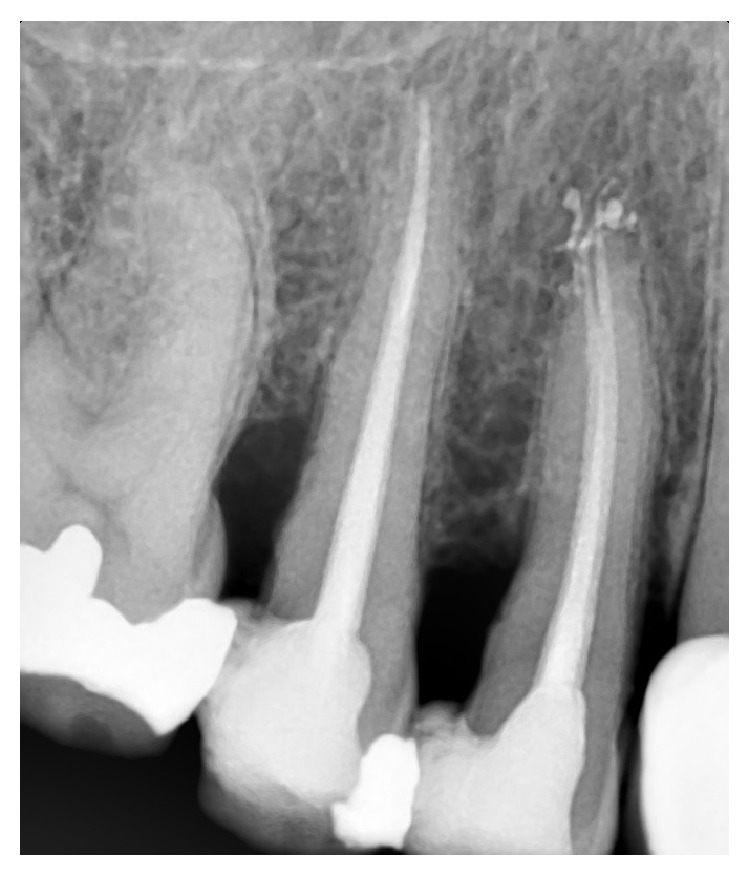
One root and one root canal.

**Figure 2:**
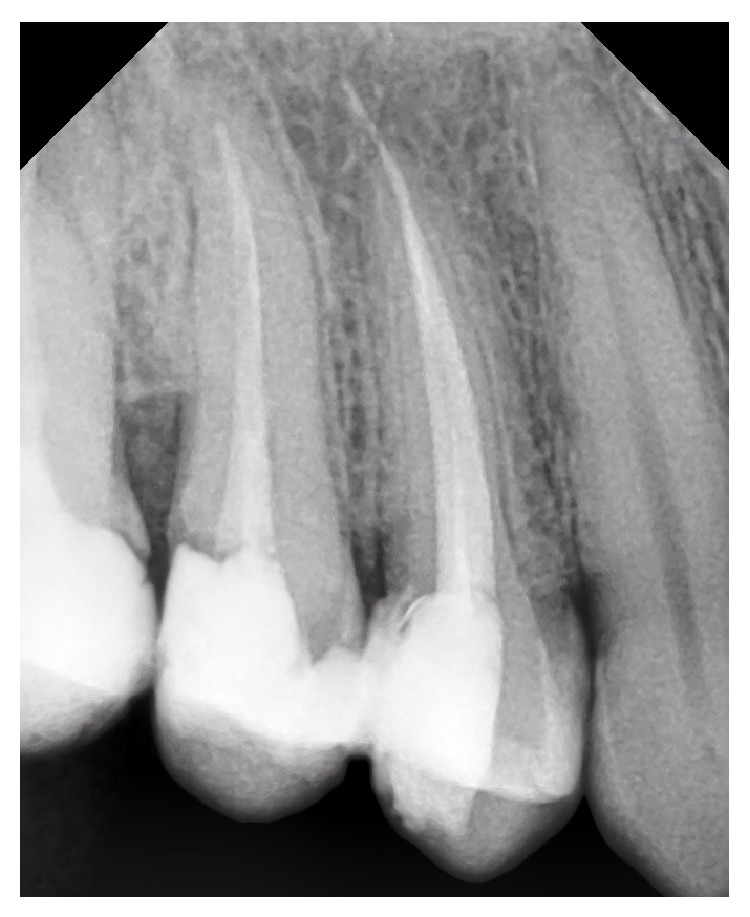
One root with two canals shared one apical foramen.

**Figure 3:**
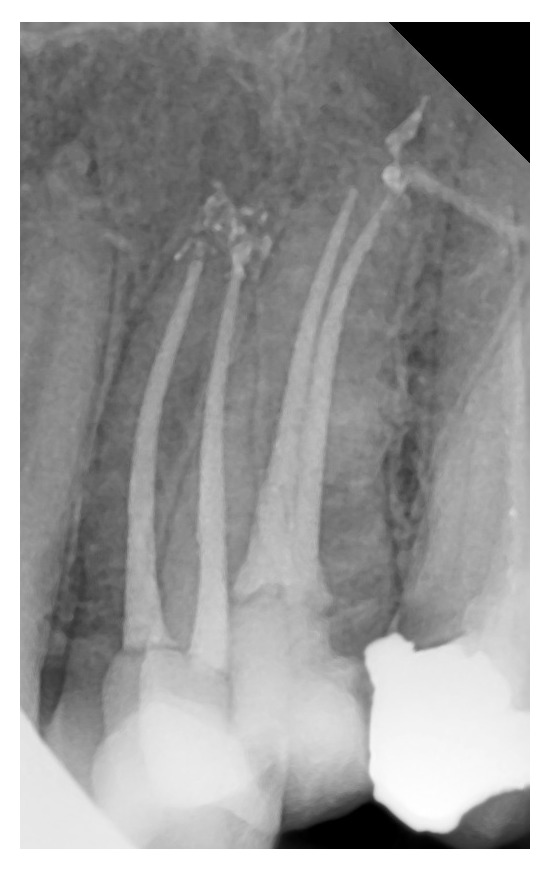
One root with two canals and two separate apical foramina.

**Figure 4:**
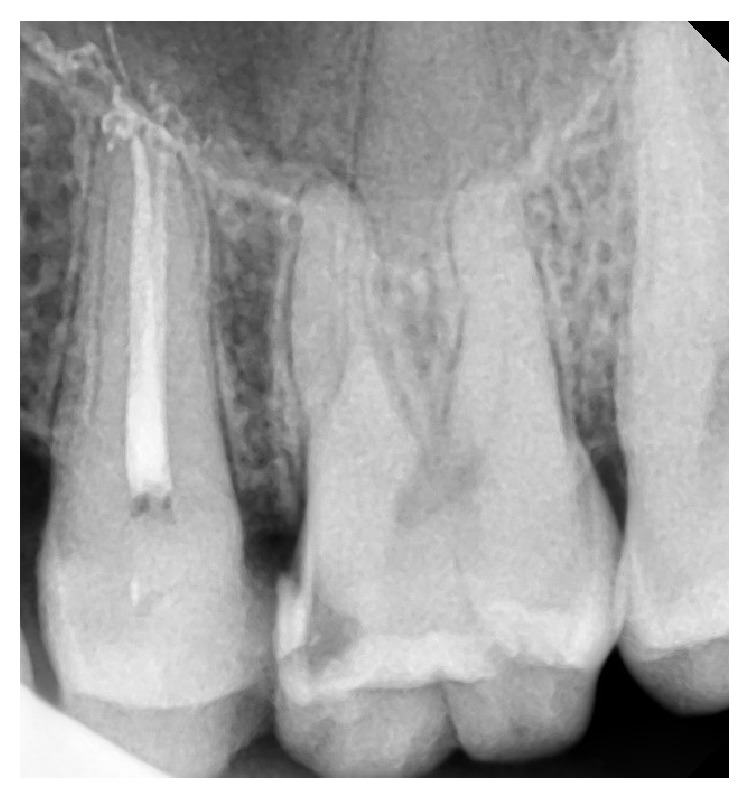
Two roots with two separate canals.

**Figure 5:**
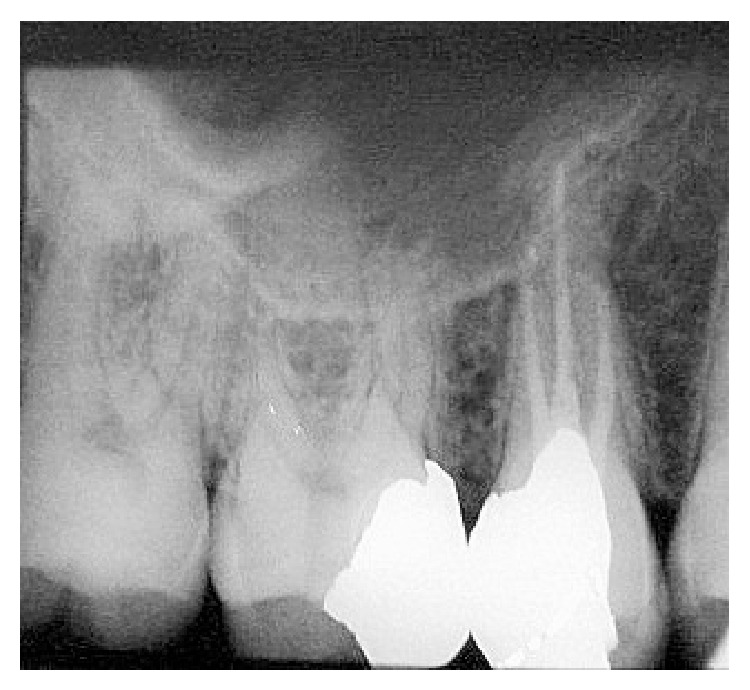
Three roots with three separate canals.

Based on Vertucci's classification of root canal morphology, 30 teeth (13.8%) had type I canal configuration (one canal with one apical foramen, Figure 1), 54 teeth (24.9%) had type II (two canal orifices end in one apical foramen, Figure 2), and 132 teeth (60.8%) had type IV (two canal orifices end in two separate apical foramina, Figures 3 and 4). One tooth (0.46%) had type VIII (three canal orifices end with three separate apical foramina, Figure 5). The incidence of two canals (types II and IV) is 85.7%.

Out of the 100 females 64% of the maxillary second premolar had one root and 36% had two roots. Regarding root canal morphology 16% of the female maxillary second premolar had type I, 32% had type II, and 52% had type IV.

Out of the 117 males 47.9% of the maxillary second premolar had one root, 51.2% had two roots, and 0.8% had three roots. Regarding root canal morphology 12% of the male maxillary second premolars had type I, 19% had type II, 68% had type IV, and 0.8% had type VIII.

## 4. Discussion 

A thorough knowledge of a common root canal morphology and its frequent variations is a basic requirement for endodontic success [1, 2]. Sometimes root canal treatment fails because the clinician fails to detect all the canals present in the tooth [3].

The number of roots and canals of the maxillary second premolar in the literature shows a wide variation [6–9]. The differences may be due to the study design (clinical versus laboratory), method of canal identification (radiographic examination, root sectioning, canal staining and root clearing, examination with SEM, or cone-beam computed tomography techniques), or true differences in the sample under investigation (racial variation) [1, 2].

Maxillary second premolar root canal system demonstrates high variability and it was the only tooth to demonstrate all eight Vertucci's canal configurations [6].

Clinically, diagnostic preoperative radiograph and its careful examination are necessary before starting root canal treatment [10–12]. Additional periapical radiographs with cone shift angulations will reveal more adequate information about root canal morphology [13]. In the present study two radiographs were taken to explore the number of roots and root canals and to take the working length of the canals during root canal treatment. One radiograph was taken at right angle and the other with 20°–40° horizontal angle cone shift. Martinez-Lozano et al. found that by varying the horizontal angle of X-ray tube 20°–40°, the number of root canals observed in maxillary first and second coincided with the actual number of canals present. Sardar et al. could identify a significantly higher number of premolars with two canals by using angled radiographs [9].

Other diagnostic measures that help in locating root canal orifices include adequate access and modification of the outline of the access cavity, exploration of the tooth's interior and exterior, and appropriate magnification and illumination [2, 3].

In this study magnifying loupes were used to help in examination of the pulp chamber floor and to identify and locate the orifices of the root canals. The use of dental loupes and dental operating microscope (DOM) provides the clinician with superior lighting and magnification improving the ability to treat cases and finding extra canals [14].

Maxillary second premolar is generally considered to have one root and one canal [2, 3, 6, 14]. In the present study only 13.8% had one root canal end in one apical foramen (type I, Figure 1). This is at variance with the earlier studies of Vertucci [7] and Kartal et al. [15] in which the maxillary second premolars were reported to have type I in 48% and 48.6%, respectively.

The present study demonstrated high incidence of two canals. The incidence of type II (two canal orifices end in one apical foramen, Figure 2) and type IV (two canal orifices end in two separate apical foramina, Figures 3 and 4) was 24.9% and 60.8%, respectively. The results of this study do not coincide with earlier studies of Vertucci et al. [6, 7] and Pécora et al. [16] who reported higher incidence of one canal and lower incidence of two canals. The results of our study are in support of Chima [17], Weng et al. [18], and Sardar et al. [9] who recorded high incidence of two root canals in the maxillary second premolar.

In the present study just one premolar (0.46%) had three roots and three canals (type VIII, Figure 5). This low incidence is consistent with other studies where the incidence ranged between 0.3% and 2% [7, 15, 16]. Three canals should be suspected clinically when the pulp chamber appears to deviate from normal configuration and does not align in its expected bucco-palatal relationship [19–21]. If the pulp chamber seems to be either triangular in shape or too large in a mesiodistal direction, more than one root canal should be suspected [22].

## 5. Conclusion 

Clinicians should be very careful when treating maxillary second premolars because of the extreme variability of the anatomy of those teeth; the risk of missing a canal in those teeth is always present.

The incidence of two canals (with either shared or separate apical foramina) is very high in the maxillary second premolars in Jordanian people. Inspection should be done for the presence of second canal whenever endodontic treatment is planned for those teeth.
